# Characterization of Gut Microbiome in the Mussel *Mytilus galloprovincialis* in Response to Thermal Stress

**DOI:** 10.3389/fphys.2019.01086

**Published:** 2019-08-22

**Authors:** Yi-Feng Li, Jia-Kang Xu, Yan-Wen Chen, Wen-Yang Ding, An-Qi Shao, Xiao Liang, You-Ting Zhu, Jin-Long Yang

**Affiliations:** ^1^International Research Center for Marine Biosciences, Ministry of Science and Technology, Shanghai Ocean University, Shanghai, China; ^2^Key Laboratory of Exploration and Utilization of Aquatic Genetic Resources, Ministry of Education, Shanghai Ocean University, Shanghai, China; ^3^National Demonstration Center for Experimental Fisheries Science Education, Shanghai Ocean University, Shanghai, China

**Keywords:** gut microbiome, thermal stress, physiological response, Illumina HiSeq sequencing, *Mytilus galloprovincialis*

## Abstract

The gut microbiota is essential for utilization of energy and nutrition and may have a role in host immunity in response to environmental shifts. The present study evaluated the temperature stress (increasing from 21 to 27°C) on gut microbiome and dynamics of the mussel *Mytilus galloprovincialis* by 16S rRNA gene sequencing with the aim of discovering the gut microbiome resilience to warming. Exposure to high temperature of 27°C significantly reduced the survival of *M. galloprovincialis* associated with increased microbial diversity of gut. The microbial communities were shifted with elevated temperature (from 21 to 27°C) and different exposure time (from day 0 to day 7) by principal coordinate analysis (PCoA). Linear discriminant analysis effect size (LEfSe) revealed that the relative abundance of *Vibrio* and *Arcobacter* presented in live animals as the top genus-level biomarkers during the initial exposure to 27°C and followed by microbiomes fluctuation with increasing exposure time at day 4 and day 7. The proliferation of opportunistic pathogens such as genus *Vibrio* and *Arcobacter* might increase host susceptibility to disease and contributed greatly to mortality. The results obtained in this study provide the knowledge on ecological adaptation for south domestication of *M. galloprovincialis* and host–bacteria interaction during temperature stress (27°C).

## Introduction

Global climate change has profound effects on marine ecosystem by altering the physiological functioning and behavior of marine organisms (Brierley and Kingsford, 2009). Variation in temperature is affecting the metabolic and respiratory rates of ectothermic animals. Presumably low stress-tolerance organisms are susceptible to disease outbreaks, which are associated with decline of population (Harvell et al., 2002; Doney et al., 2012). Although a shift of biogeographic distribution has occurred in mobile marine organisms (Beaugrand et al., 2002), the sedentary organisms such as mussel and oyster are more prone to mass mortality in summer (Myrand et al., 2000; Malham et al., 2009).

The mussel *Mytilus galloprovincialis* is an important commercial aquaculture species and mainly cultured in China and Europe (Lazo and Pita, 2012). *M. galloprovincialis* is widely distributed along the northern shores of Liaodong and Shandong peninsulas in north of China and the seawater temperature reaches to 25°C in these habitats during summer season (Xiao et al., 2005; Lin et al., 2011). Recently, *M. galloprovincialis* has been cultured in the coast of Zhoushan (Zhejiang Province, China), where is a natural habitat of the mussel *M. coruscus* (Wang et al., 2012; Yang et al., 2014). The average of surface seawater temperature was 27°C during the summer season in Zhoushan (Li et al., 2018) and it might result in the reduction of fitness and increment of mortality.

The gut of marine animals shelter species-rich microbial communities, which provides biological function for the host such as competition with pathogens and disease resistance (Gatesoupe, 1999). Previous evidences showed that exposure to high water temperature of 31°C altered the *M. coruscus* gut microbiome by facilitating the proliferation of opportunistic bacteria in mussel adults (Li et al., 2018). Therefore, the disturbance of gut microbiota may reflect the healthy status of the host. To determine physiological responses of *M. galloprovincialis* acclimating to seawater temperature increasing from 21 to 27°C, the effects of temperature stress (27°C) on *M. galloprovincialis* gut microbiota using Illumina Hiseq sequencing of 16S rRNA gene was analyzed. The change of microbial community was determined in response to temperature stress (27°C). The knowledge obtained in this study will provide the information for south domestication and mariculture of *M. galloprovincialis* in changing environment.

## Materials and Methods

### Ethics Statement

The experimental protocol for mussel acclimation and experimentation was approved by the Animal Ethics committee of Shanghai Ocean University, Shanghai, China.

### Biological Material

Wild population of juvenile *M. galloprovincialis* (1.8 ± 0.3 cm in shell length and 1.4 ± 0.3 cm in shell width) were collected at Lvshunkou district (38°45′ N, 121°13′ E), Dalian, Liaoning Province, China, in October 2018. The mussels were reared in 10 L polycarbonate tanks (30 mussels/tank) containing seawater (salinity: 30) with aeration at 21°C, which was the average seawater temperature in mussel collection site. The seawater was renewed daily. The algae *Platymonas helgolandica* var. *tsingtaoensis* and *Isochrysis zhanjiangensis* were supplied to the mussel as food source as previous described (Li et al., 2018). Mussels were acclimated for 1 week before the start of experiment at 21°C.

### Experimental Design and Gut Sampling

Mussels were exposed for 7 days to determine the effects of temperature stress on the gut microbiota of the mussel *M. galloprovincialis*. The mussels (*n* = 300) were kept in triplicate 15-L polycarbonate tanks (0.12 m^2^) at 21 ± 1°C (control) or 27 ± 1°C (treatment) and fed daily with *I. zhanjiangensis* or *P. helgolandica* (algal cell density of 8 × 10^4^ cells/mL). For treatment group at 27°C, an increase of temperature from 21 to 27°C was performed by gradually increased 1°C/day to minimize heat shock (Webster et al., 2011). The mussels were sampled at 0, 4, and 7 days in control and treatment groups (20 individuals per replicate tank).

The seawater was renewed every day in each treatment groups. The tanks were frequently checked for removing the dead mussels when identified and the cumulative number of deaths were calculated on a 24 h basis. The gut samples from live mussels were obtained by dissecting mussels with tweezer and scissor. Each gut sample was collected from 10 individual juvenile mussels and stored at −80°C prior to bacterial DNA extraction.

### Bacterial DNA Extraction and PCR Amplification

Total DNA was isolated from the stored gut samples (*n* = 3) using a MOBIO PowerSoil^®^ DNA Isolation Kit (MOBIO Laboratories, Carlsbad, CA, United States) and following the protocol provided by manufacturer. The DNA concentration and purity were checked using the NanoDrop One (Thermo Fisher Scientific, Waltham, MA, United States) and followed by the amplification of the 16S ribosomal RNA gene (V3–V4 region) using universal bacterial primers, 338F (5′-ACTCCTACGGGAGGCAGCA-3′) and 806R (5′-GGACTACHVGGGTWTCTAAT-3′) tagged with 12 bp barcode. PCR reactions, containing 25 μL 2× Premix Taq (Takara Biotechnology, Dalian, Co., Ltd., China), 1 μL each primer (10 mM) and 3 μl DNA (20 ng/μL) template in a volume of 50 μL, were amplified by thermocycling: 5 min at 94°C for initialization; 30 cycles of 30 s denaturation at 94°C, 30 s annealing at 52°C, and 30 s extension at 72°C; followed by 10 min final elongation at 72°C.

### Illumina HiSeq Sequencing

The PCR products were examined on 1% agarose gel electrophoresis. PCR products were mixed in equidensity ratios using the GeneTools analysis software (Version4.03.05.0, SynGene) prior to the purification of the mixture PCR products using E.Z.N.A. gel extraction kit (Omega, United States). Sequencing libraries of each group were generated using NEBNext^®^ Ultra^TM^ DNA Library Prep Kit for Illumina^®^ (New England Biolabs, Ipswich, MA, United States) following manufacturer’s instructions. The quality of sequencing libraries was checked on the Qubit^®^ 2.0 Fluorometer (Thermo Fisher Scientific, Waltham, MA, United States) and Agilent Bioanalyzer 2100 system (Agilent Technologies, Waldbronn, Germany). Sequencing was performed on an Illumina Hiseq 2500 platform and 250 bp paired-end reads were generated (Guangdong Magigene Biotechnology, Co., Ltd., Guangzhou, China). Raw sequences have been submitted to the NCBI sequence read archive database under the accession number: SRP197453.

### Statistical and Bioinformatics Analysis

The statistical analyses of microbial diversity indices (Chao1, Shannon, and Simpson) were performed by Wilcoxon/Kruskal–Wallis test after tested for normality (Shapiro–Wilk test) and homogeneity (O’Brien test) using JMP^TM^ software (SAS Institute, Shanghai, China) and *P*-value < 0.05 was considered significantly different.

Trimmomatic (V0.33^1^) was used to remove the low quality sequences, reads with N and sequence < 100 bp long of the raw reads. FLASH (V1.2.11^2^) was used to assemble the filtered sequences followed by removing barcodes and primers by Mothur software (V1.35.1^3^) for obtaining the clean Tags. The OTUs were determined using a similarity threshold of 97% by the Usearch software (version 10.0.^4^). Taxonomic annotation of 16S rRNA gene sequence was determined with the RDP classifier^5^ against the database of Silva (Release132^6^) with a confidence threshold of 0.5. R software was performed to construct the histogram, heatmap, principal coordinate analysis (PCoA), the diversity indices of Chao1 Simpson and Shannon for analyzing the bacterial community composition. Linear discriminant analysis effect size (LEfSe) was performed to determine differences of the relative abundance of bacteria at all taxonomic levels between the treatment and control. The Kruskal–Wallis test was conducted to identify bacterial taxa that are significantly different in relative abundance among different samples. The linear discriminant analysis (LDA) identifies the effect size with which these taxa differentiate the samples with thresholds of a log-transformed LDA score of 2.0.

## Results

### *M. galloprovincialis* Survival

**FIGURE 1:**
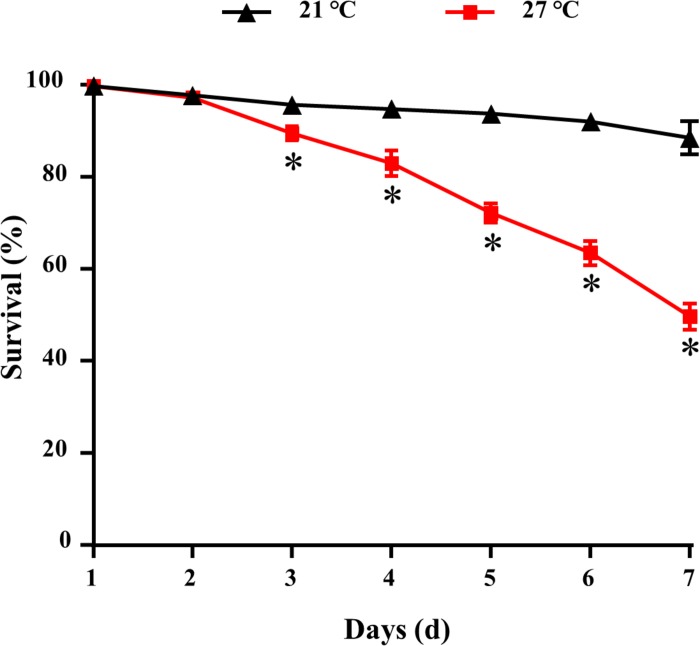
Survival for *M. galloprovincialis*. ^∗^*P* < 0.05.

The survival of mussels cultured at 21 and 27°C for 7 days were shown in Figure 1. Significant difference was observed between control and warm acclimated mussels after 3 days exposure (*P* < 0.05). After 7 days, the lowest survival rate (50 ± 2%) was observed in the mussels exposed to higher temperature at 27°C.

### Gut Microbiome Analysis

Globally 1152 operational taxonomic units (OTUs) were identified from the gut samples. At a 3% dissimilarity level, Good’s coverage showed that 99.7 to 99.9% OTUs were identified for all of the groups. Based on OTUs at 3% dissimilarity, the rarefaction curve of each group tended to approach the saturation plateau (Supplementary Figure S1).

### Composition of Gut Microbiota at Phylum Level

**FIGURE 2:**
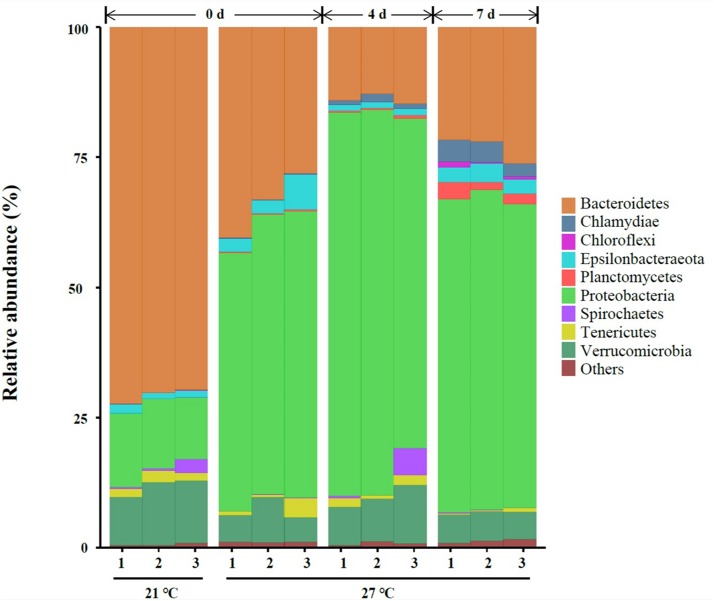
Relative abundance of bacterial communities at the phylum level of gut samples. Three replicates are labeled with the numbers 1, 2, and 3.

A total of nine different phyla in all samples with an abundance of > 1% were characterized, and the abundance of nine phyla < 1% were all classified as “others” (Figure 2). Three dominant phyla Bacteroidetes, Proteobacteria and Verrucomicrobia were present in gut samples, which accounted for 87.2–95.9% of total reads (Figure 2 and Supplementary Table S1).

High temperature (27°C) significantly increased the relative abundance of Chlamydiae, Epsilonbacteraeota, Planctomycetes, and Proteobacteria on day 0. A significant reduction of the relative abundance of Bacteroidetes, Spirochaetes, and Verrucomicrobia were observed in warm acclimated mussels compared to control mussels on day 0 (*P* < 0.05, Supplementary Table S1). At 27°C, the relative abundance of Chlamydiae, Planctomycetes, and Proteobacteria were significantly increased in mussels collected on day 4 compared to day 0 (*P* < 0.05, Supplementary Table S1). Warm acclimated mussels significantly reduced the relative abundance of Bacteroidetes and Epsilonbacteraeota in mussels collected on day 4 compared to day 0 (*P* < 0.05, Supplementary Table S1). In a comparison between day 4 and day 7, the relative abundance of Bacteroidetes, Chlamydiae, Chloroflexi, Epsilonbacteraeota, and Planctomycetes were significantly increased, and this was accompanied by a significant reduction in the relative abundance of Proteobacteria and Verrucomicrobia (*P* < 0.05, Supplementary Table S1).

### Composition of Gut Microbiota at Genus Level

**FIGURE 3:**
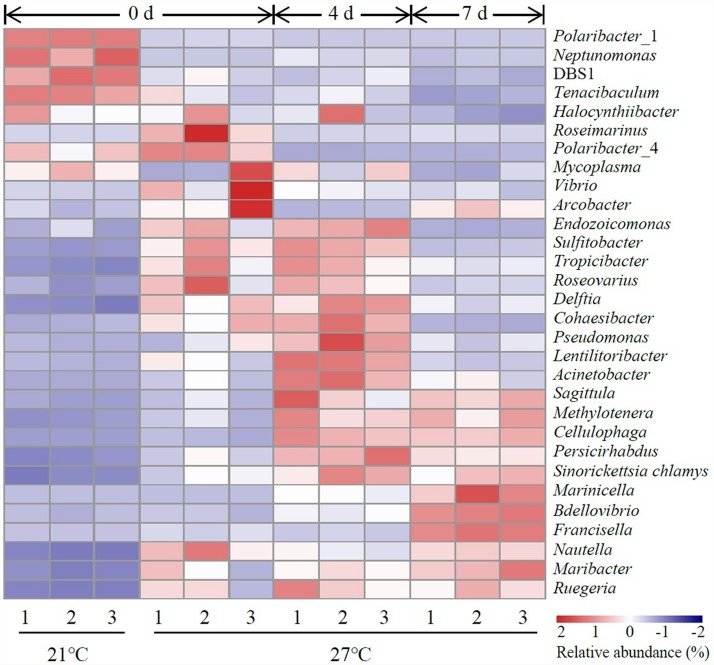
Heatmap revealing the top 30 bacterial genera (%) of gut samples. Three replicates are labeled with the numbers 1, 2, and 3.

The top 30 abundant genera were constructed for comparative analysis (Figure 3). *Polaribacter*_1 was a dominant genus in control groups which accounted for 57.8–59.6% of total reads (Supplementary Table S2). *Vibrio* was a dominant genus in treatment groups at 27°C on day 0 and the relative abundance of *Vibrio* was significantly higher in treatment groups (27°C) than control groups (21°C) on day 0 (*P* < 0.05, Supplementary Table S2). The relative abundance of *Acinetobacter*, *Bdellovibrio*, *Cellulophaga*, *Lentilitoribacter*, *Marinicella*, *Methylotenera*, *Neptunomonas*, *Persicirhabdus*, *Pseudomonas*, and *Sinorickettsia_chlamys* were significantly increased in treatment groups on day 4 compared to day 0 and associated with a significant reduction in the relative abundance of *Arcobacter*, *Nautella*, *Polaribacter*_1, and *Roseimarinus* (*P* < 0.05, Supplementary Table S2). In the comparison groups between day 4 and day 7, continuous exposure to warm temperature favored proliferation of a dominant genus *Marinicella* as well as other genera such as *Arcobacter*, *Bdellovibrio*, *Francisella*, *Maribacter*, *Nautella*, and *Roseimarinus* (*P* < 0.05, Supplementary Table S2).

**FIGURE 4:**
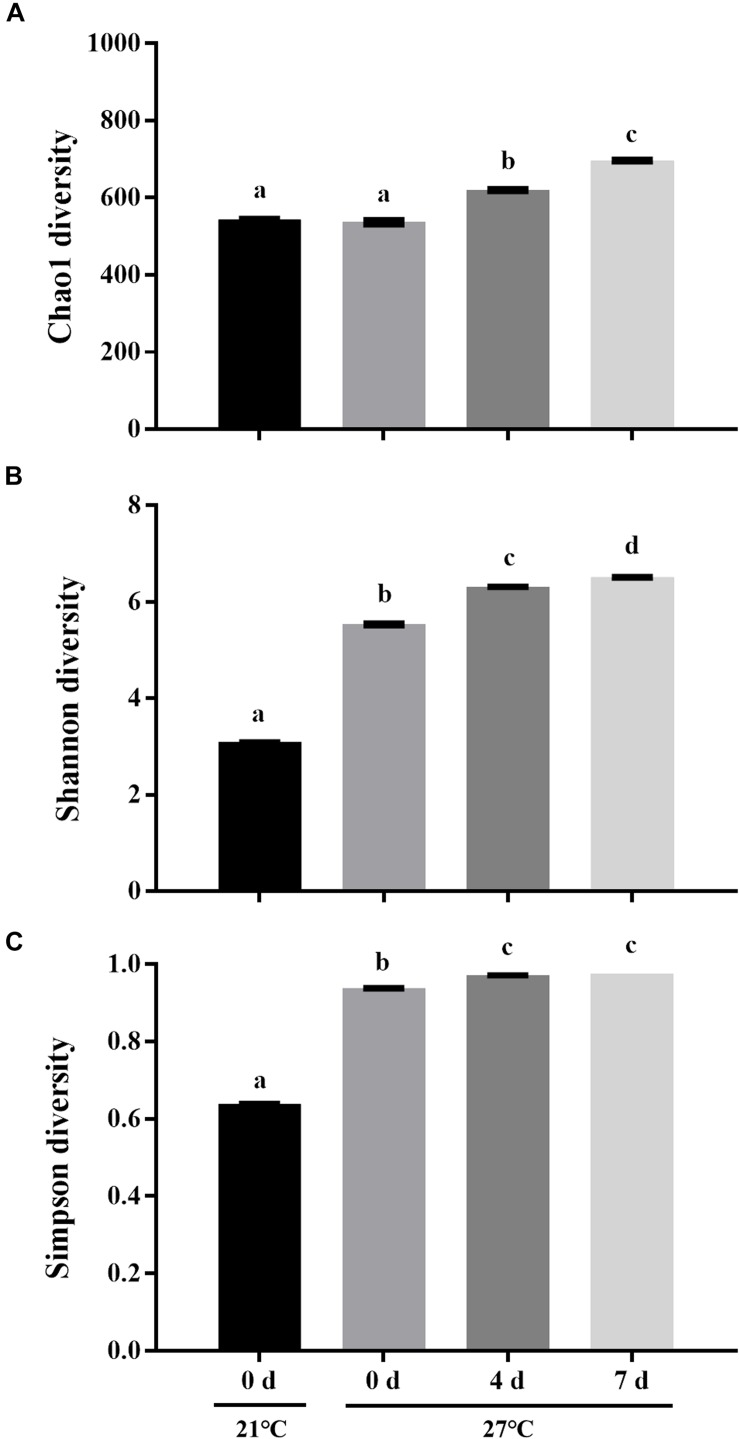
Microbial diversity indices of Chao1 **(A)**, Shannon **(B)**, and Simpson **(C)**. Data are the mean ± SE (*n* = 3). Different letters represent significant differences (*P* < 0.05).

**FIGURE 5:**
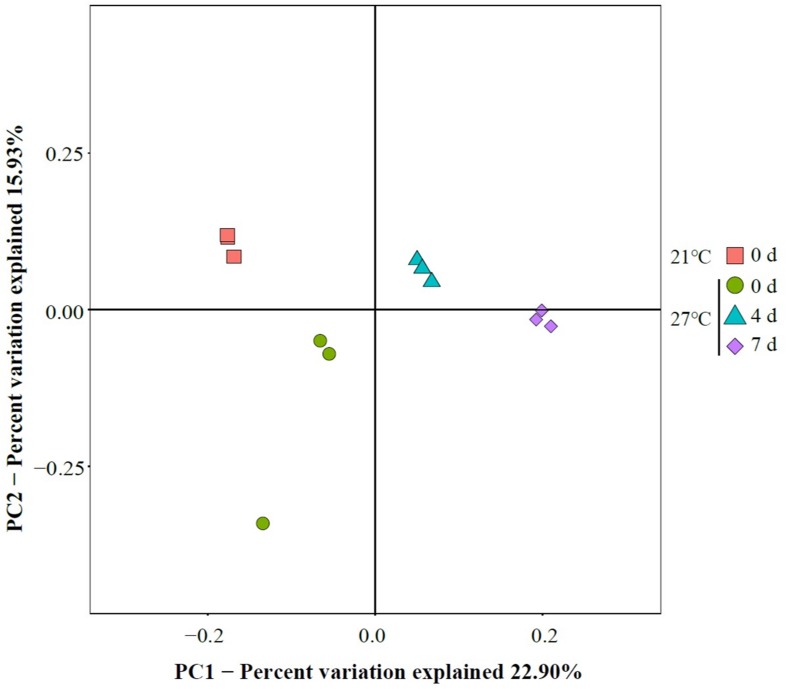
Principal coordinate analysis (PCoA) of gut microbiome.

To characterize the microbial diversity of the gut samples, alpha diversity metrics such as total species abundance index (Chao1, Figure 4A) and species diversity indices (Shannon, Figure 4B; Simpson, Figure 4C) were analyzed. No significant difference of Chao1 index was observed in the treatment group at 27°C relative to control groups (*P* > 0.05). Warm temperature significantly increased Chao1 index with increasing exposure time (*P* < 0.05). Exposure to high temperature significantly increased the Shannon index on day 0 relative to control groups (*P* < 0.05). A rise of the Shannon index value was observed in groups at 27°C with increasing exposure time. Similarly, the Simpson index revealed significantly higher diversity in the treatment groups at 27°C relative to control groups (*P* < 0.05). PCoA analysis revealed that temperature shaped the microbial communities by separating the control and treatment groups on day 0 with the groups on day 4 and 7 at 27°C by PC1, which made up > 22.90% of the variance (Figure 5). Clear dissimilarities were observed in the treatment groups within various exposure times.

### Unique Biomarkers Detected in the Mussel Gut

**FIGURE 6:**
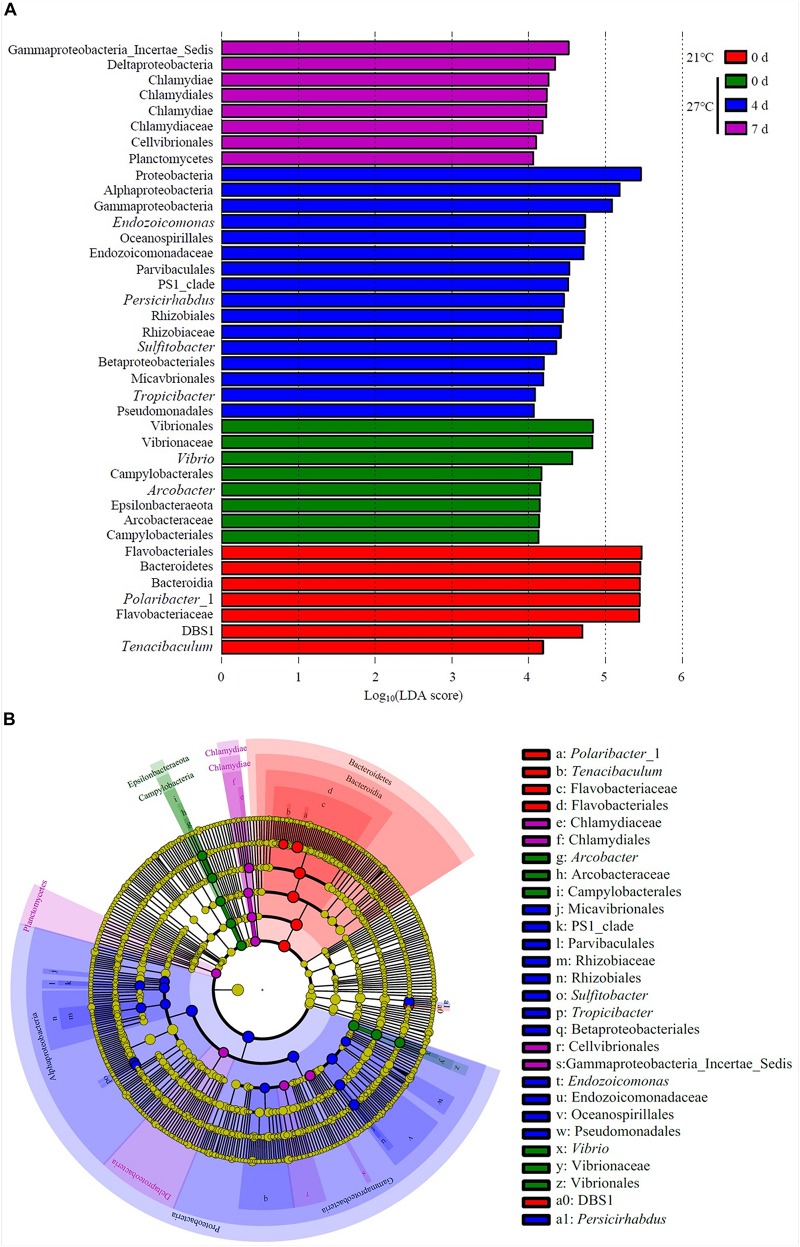
Unique community composition of biomarkers in mussel gut. **(A)** Bar chart showing the log-transformed LDA scores of bacterial taxa identified by LEfSe analysis. A log-transformed LDA score of 2 was used as a threshold for identification of significant taxa, 39 taxa were identified by LEfSe analysis and are shown (Supplementary Table S3). **(B)** Cladogram showing the phylogenetic relationships of 47 bacterial taxa revealed by LEfSe.

LEfSe analysis revealed that *Polaribacter*_1 and *Tenacibaculum* were the top genus-level biomarkers distinguished the control group at 21°C from all other groups (Figure 6 and Supplementary Table S3). The gut samples collected in treatment groups at 27°C on day 0 were distinguished from all other groups by the relative abundance of *Vibrio* and *Arcobacter*. *Endozoicomonas*, *Persicirhabdus*, *Sulfitobacter*, and *Tropicibacter* were the top genus-level biomarkers in treatment groups on day 4 compared to all other groups.

## Discussion

The present study provides the knowledge of mussel physiological responses to temperature stress. The results showed that increased mortality of the mussel *M. galloprovincialis* with raising seawater temperature from 21 to 27°C. Temperature affected microbial communities and dynamics in mussel gut as determined by 16S rRNA gene sequencing in live mussels and higher microbial diversity was found in live gut samples within short-term exposure to warm temperature. PCoA analysis revealed that the bacterial communities were affected by continuous heat stress. Unique species biomarkers determined by LEfSe analysis were presented good indicators of healthy status.

The geographic distributions of individual variation are determined by both adaptation and phenotypic plasticity in different latitudinal gradient, which is related to thermal tolerance range in species level (Sorte et al., 2011). Temperature stress was reported as a key factor in summer mortality syndrome (SMS) of marine bivalves (Myrand et al., 2000; Xiao et al., 2005; Malham et al., 2009). Generally, *M. edulis* was unable to tolerate temperatures above 28.5°C (Chapple et al., 1998). Laboratory trials demonstrated that temperature stress (31°C) had been directly linked to adult mussel *M. coruscus* mortality (29 and 49%) when fed two different diets, whereas exposure to 27°C for 3 days caused nearly 10% mortality rate (Li et al., 2018). In the present study, *M. galloprovincialis* exposed to 27°C for 7 days had contributed to 50% mortality, which indicates that the homeostasis of mussels held at 27°C may be disrupted. The divergent geographic distribution of *M. galloprovincialis* and *M. coruscus* might indicate that thermal tolerance could be an important physiological limit.

Warm temperature significantly increased microbial diversity in gut microbiota of mussels as indicated by Chao1, Shannon, and Simpson indexes. However, a significant reduction of microbial diversity was observed in gut microbiome of live mussel *M. coruscus* under heat stress (Li et al., 2018). This discrepancy may be explained by the alteration of community structure together with species-rich microbiome contributed to the high resilience threshold of animals under adverse conditions (Lozupone et al., 2012). Species diversity is strongly positively correlated with ecosystem stability which enables the community less susceptible to perturbation (Kühsel and Blüthgen, 2015; Sommer et al., 2017). It was reported that warm temperature impaired health condition in oysters associated with decreased microbial diversity (Lokmer and Wegner, 2015). It remains to be established whether the variation of microbial community diversity is caused by the mussel physiological responses to heat stress or a direct effect by temperature *per se*.

In the present study, PCoA analysis revealed that warm temperature altered major bacterial species. This is supported by previous study that a separation was observed between the gut microbiota of live mussel *M. coruscus* challenged by high temperature (Li et al., 2018). Furthermore, the results of present study extended our previous study by showing continuously exposure at 27°C reduced the similarity of community composition, and the shift of core microbial community over time could possibly be an indicator of high mortality. Similarly, the microbial dynamics and community composition in the oyster haemolymph was influenced by heat stress and suggested that instability of microbiome might contribute to high mortality (Lokmer and Wegner, 2015). Although the exact mechanisms of host–bacteria interaction are elusive, shifts of bacterial communities in gut may closely relate to host’s physiology (Chen et al., 2018).

The present study revealed that Bacteroidetes, Proteobacteria, and Verrucomicrobia were three dominant bacterial phyla in *M. galloprovincialis* which differ of the previous study where Bacteroidetes, Proteobacteria and Firmicutes were prevalent in *M. coruscus* gut (Li et al., 2018). Bacteroidetes and Proteobacteria were abundantly found in guts of small abalone *Haliotisdiversicolor* (Zhao et al., 2018), crab *Callinectes sapidus* (Givens et al., 2013) and sea urchin *Lytechinus variegatus* (Hakim et al., 2015). Verrucomicrobia is commonly found in soil and marine environment (Freitas et al., 2012) as well as gut samples from mammalian (Dubourg et al., 2013), amphibian (Kohl et al., 2013), and bivalve (King et al., 2012). In the case of the mammalian, the relative abundance of Verrucomicrobia is suggested to be associated with the adaptive immunity (Zhang et al., 2015). It is noteworthy that *M. galloprovincialis* harboring a high proportion of microbes belonging to the phyla Verrucomicrobia even under heat stress.

An increase in water temperature (from 21 to 27°C) reduced the abundances of genus *Polaribacter* belong to the phylum Bacteroidetes. *Polaribacter* species have been detected in marine environment such as seawater (Yoon et al., 2006; Fukui et al., 2013). This species have been isolated from diatom phytoplankton blooms, which may be involved in the decomposition of sulfated polysaccharides (Xing et al., 2015). In the gut of *M. galloprovincialis* exposed to 27°C on day 0, there was a high abundance of the genus *Vibrio* and *Arcobacter* as revealed by LEfSe analysis. *Vibrio* species is ubiquitous microbes in marine environment. Warm temperature favored the proliferation of *Vibrio* and contributed to the mass mortality in bivalve shellfish aquaculture (Elston et al., 2008; Le Roux et al., 2016). Previous study on the oyster indicated that moribund oysters were dominated by the genus *Arcobacter*, which suggested as opportunistic pathogens (Lokmer and Wegner, 2015). The genome of *Endozoicomonas* bacteria showed their enrichment genes related to metabolic function such as utilization of carbohydrates and the supply of proteins to their host (Neave et al., 2017). In addition, *Endozoicomonas* species seems to have symbiotic relationships with the host by producing antimicrobial substance to deter potential invading microbes (Bourne et al., 2008). The genus *Endozoicomonas* dominated in gut of *M. galloprovincialis* exposed to 27°C on day 4, indicating those microorganisms were suspected to play a crucial role in maintaining health.

The present study showed that temperature was a key factor determining gut microbial community in *M. galloprovincialis*. The responses to heat stress were through the shifts of bacterial communities. Proliferation of opportunistic pathogens such as genus *Vibrio* and *Arcobacter* may increase host susceptibility to disease in response to heat stress and contributed to the mass mortality in mussel *M. galloprovincialis* aquaculture. The shifts of those opportunistic pathogens are suggested to be good indicators of mussel’s physiology. The results obtained in this study provide the knowledge on mariculture of *M. galloprovincialis* associate with ecological adaptation for south domestication in changing environment and host–bacteria interaction during temperature stress.

## Data Availability

Publicly available datasets were analyzed in this study. This data can be found here: Raw sequences have been submitted to the NCBI sequence read archive database under the accession number: SRP197453.

## Ethics Statement

The experimental protocol for mussel acclimation and experimentation was approved by the Animal Ethics committee of Shanghai Ocean University, Shanghai, China.

## Author Contributions

J-LY, XL, and Y-FL conceived and designed the experiments. J-KX, Y-WC, W-YD, and A-QS performed the experiments. Y-FL, J-KX, XL, and J-LY analyzed the data. Y-FL, Y-TZ, J-LY, and XL wrote the manuscript. All authors reviewed the manuscript.

## Conflict of Interest Statement

The authors declare that the research was conducted in the absence of any commercial or financial relationships that could be construed as a potential conflict of interest.

## Footnotes

1 http://www.usadellab.org/cms/?page=trimmomatic

2 https://ccb.jhu.edu/software/FLASH/

3 http://www.mothur.org

4 http://www.drive5.com/usearch/

5 http://rdp.cme.msu.edu/

6 http://www.arb-silva.de

## Supplementary Material

FIGURE S1 Operational taxonomic units (OTUs) represented by rarefaction curve of all gut samples.

TABLE S1 Relative abundance (in%) of the sequences in gut assigned to different phyla.

TABLE S2 Relative abundance (in%) of the sequences in gut assigned to different genera.

TABLE S3 All bacterial taxa (log-transformed LDA > 2) identified by LEfSe analysis of *M. galloprovincialis*.
