# Supplementary figures and images for: Characterization of Gut Microbiome in the Mussel Mytilus galloprovincialis in Response to Thermal Stress
Source: Front Physiol. 2019 Aug 22;10:1086. doi: 10.3389/fphys.2019.01086 (PMC6714297; doi:10.3389/fphys.2019.01086)

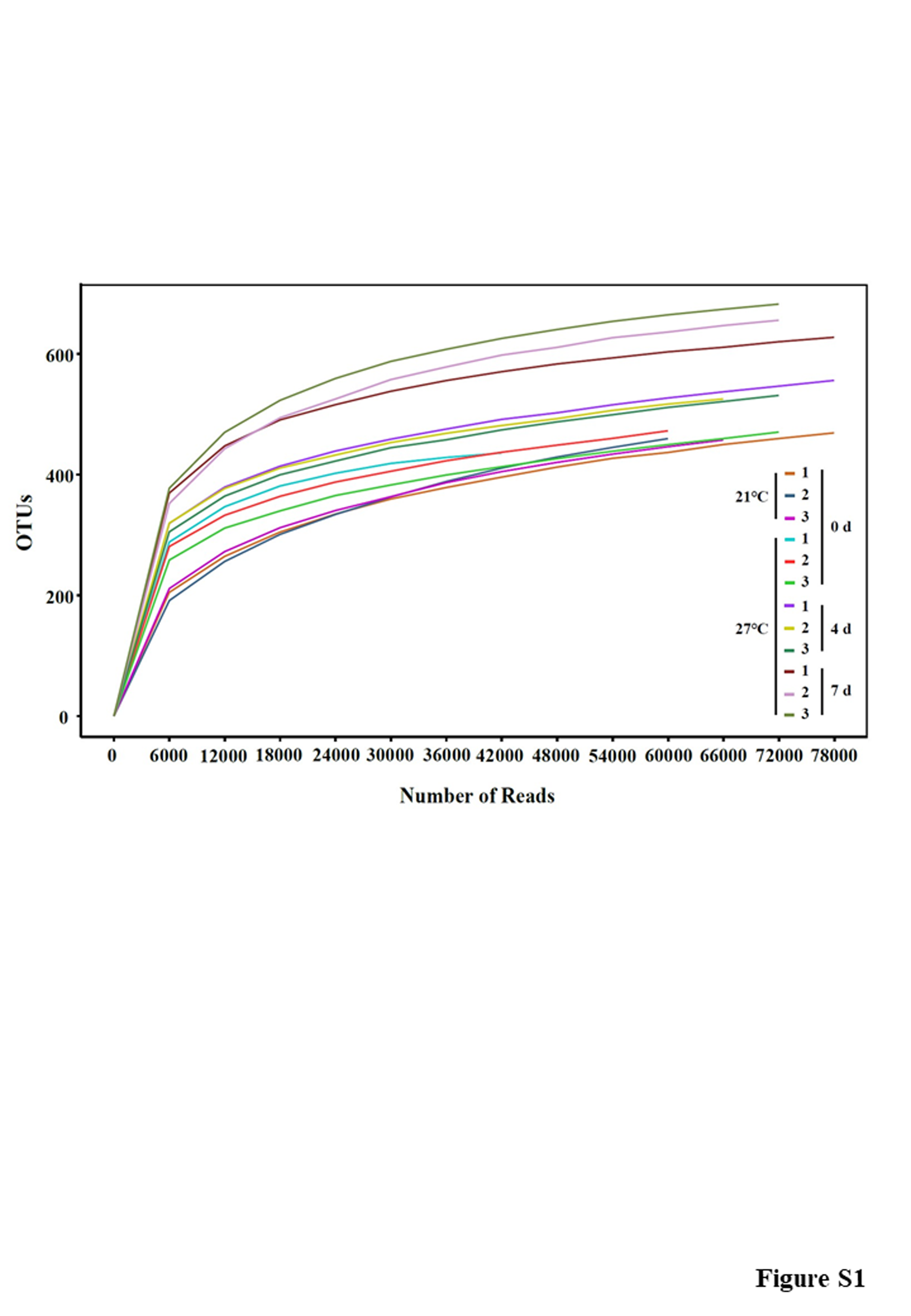

Supplement: FIGURE S1 — Operational taxonomic units (OTUs) represented by rarefaction curve of all gut samples. [file Image_1.TIF]
